# Valacyclovir-induced neurotoxicity and nephrotoxicity in an elderly patient with a history of nephrectomy: a case report

**DOI:** 10.1186/s12882-025-03941-7

**Published:** 2025-05-30

**Authors:** Sondos Badran, Sylvia Li, Sabrina Nguyen, Johnny S. Randhawa, Abdulrahman Badran, Farbod Farmand

**Affiliations:** 1 Department of Internal Medicine, Arrowhead Regional Medical Center, Colton, CA 92324 USA; 2 California University of Science and Medicine, Colton, CA 92324 USA; 3 Western University of Health Sciences, Pomona, CA 91766 USA

**Keywords:** Valacyclovir, Nephrotoxicity, Neurotoxicity, Nephrectomy

## Abstract

**Background:**

Valacyclovir is a prodrug of acyclovir, both of which are commonly used in the treatment of varicella zoster and herpes simplex viruses. Its mechanism as a guanosine analog antiviral inhibits DNA polymerase via chain termination. It has selective action in infected cells with minimal effect on host cells, lending to fewer side effects, and is a well-tolerated medication often chosen for its better oral bioavailability over acyclovir. Its side effect profile includes thrombotic thrombocytopenic purpura (TTP), gastrointestinal symptoms such as nausea, and increased transaminases. Common adverse effects include headache, nausea, and vomiting. A rare adverse effect of valacyclovir is acute kidney injury (AKI) due to obstructive crystal-induced nephropathy, tubular dysfunction, or tubulointerstitial nephritis. To date, no case report has cited normal dosing of valacyclovir causing acute kidney injury in a patient status post nephrectomy.

**Case present:**

We present a 71-year-old female with history of chronic kidney disease (CKD), left nephrectomy due to renal agenesis and nephrolithiasis, type 2 diabetes mellitus, hypertension, and hyperlipidemia who presented to the emergency department due to visual and auditory hallucinations after taking two days of valacyclovir for shingles. Laboratory work demonstrates elevated creatinine at 6.19 mg/dL. Patient was monitored throughout hospitalization with cessation of valacyclovir. Patient’s creatinine down-trended.

**Conclusion:**

This case report demonstrates a rare case of acute kidney injury due to an exaggerated response to valacyclovir-induced crystal nephropathy, presenting in tandem with valacyclovir-induced neurotoxicity, which has only been described in three case reports. This case highlights the importance of careful history and physical exam to facilitate a more accurate and timely diagnosis, especially in patients with a history of nephrectomy.

## Background

Valacyclovir is a prodrug of acyclovir commonly used in the treatment of varicella zoster and herpes simplex virus [1]. Its mechanism as a guanosine analog antiviral inhibits DNA polymerase via chain termination. Valacyclovir has a greater bioavailability and longer half-life than acyclovir. This allows for less frequent dosing of valacyclovir. Adverse reactions of valacyclovir include acute kidney failure (AKI) and encephalopathy [2]. Hospital admission rate among patients with AKI who received valacyclovir is 0.27% [3]. The incidence of neurotoxicity caused by valacyclovir is less than 1% [4]. Here we discuss a 71-year-old female who had a normal creatinine and mental status one week prior to presenting at the hospital for acute encephalopathy. She was subsequently also found to have an acute kidney injury, which developed after being prescribed valacyclovir one gram three times a day for seven days by her ophthalmologist for ocular shingles.

### Case presentation

A 71-year-old Hispanic female with a history of chronic kidney disease (CKD) stage 3a, left nephrectomy due to renal agenesis and nephrolithiasis, type 2 diabetes mellitus, hypertension and hyperlipidemia presented to the emergency department with auditory and visual hallucinations. A week prior to presentation, she developed blisters over her eyes and face on the left side. Patient was seen by an ophthalmologist who prescribed her a seven day course of oral valacyclovir, 1 gram three times a day for shingles for one week, which is the standard for herpes ophthalmicus. After taking the medication for two days, the patient started experiencing dizziness, nausea, vomiting, and diarrhea. Three days into her treatment she began hallucinating. Patient’s home medications included metformin 1000 mg twice a day, amlodipine 5 mg daily, benazepril 40 mg daily, and rosuvastatin 10 mg nightly.

In the emergency department, her vital signs were stable, including her blood pressure of 121/76 mmHg, a heart rate of 75 beats per minute, a temperature of 98.9 F, a respiratory rate of 18 breaths per minute, and an oxygen saturation of 98% on room air. On the physical exam, the patient was alert and oriented, answering questions appropriately. However, at times, the patient was seen responding to internal stimuli. Patient’s left eye had crusted lesions over the left V1 dermatome distribution. Initial comprehensive metabolic panel revealed a sodium of 131 mmol/L, chloride of 95 mmol/L, bicarbonate of 18 mmol/L, blood urea nitrogen (BUN) of 56 mg/dL and creatinine of 6.19 mg/dL, estimated glomerular filtration ratio (eGFR) 6.8 ml/min/1.73m^2^, lactate 1.8 mmol/L (Table 1). Computed tomography (CT) of the head without contrast was ordered to rule out any hemorrhagic lesions due to a change in mentation, which was negative. Ophthalmology was consulted and recommended renally dosing valacyclovir to 500 mg once daily to complete a 7-day course. Additionally, bacitracin ointment twice per day and artificial tears every 2 hours were recommended for ocular varicella zoster virus. However, due to creatinine function worsening in comparison to her previous laboratory work one month ago, valacyclovir was not started inpatient. The patient was admitted to the hospital for further work up of her elevated creatinine and altered mental status.

**Table 1:** Initial laboratory values on presentation

Laboratory study	Reference values	Measured values
Hemoglobin	11.5–15.5 g/dL	13.4
Hematocrit	36–46%	40
Platelet	120–360 × 10^3^ /uL	218
Sodium	135–148 mmol/L	131
Potassium	3.5–5.5 mmol/L	5.5
Chloride	98–110 mmol/L	95
CO2	24–34 mmol/L	18
Blood urea nitrogen	8–20 mg/dL	56
Creatinine	0.50–1.50 mg/dL	6.19
Estimated glomerular filtration ratio	> 90 ml/min/1.73m^2^	6.8
Glucose	65–125 mg/dL	122
Calcium	8.5–10.5 mg/dL	10.1
Total Bilirubin	0.0-1.2 mg/dL	0.5
Alkaline Phosphatase	35–125 U/L	69
AST	5–40 U/L	17
ALT	5–40 U/L	13
Total Protein	6.0–8.0 d/dL	6.6
Albumin	3.5–4.9 g/dL	4.1
Lactate	1-1.8 mmol/L	1.8
Urine eosinophil	negative	Negative
Urinalysis	Variable	Trace protein Trace leukocyte esterase

**Fig. 1:**
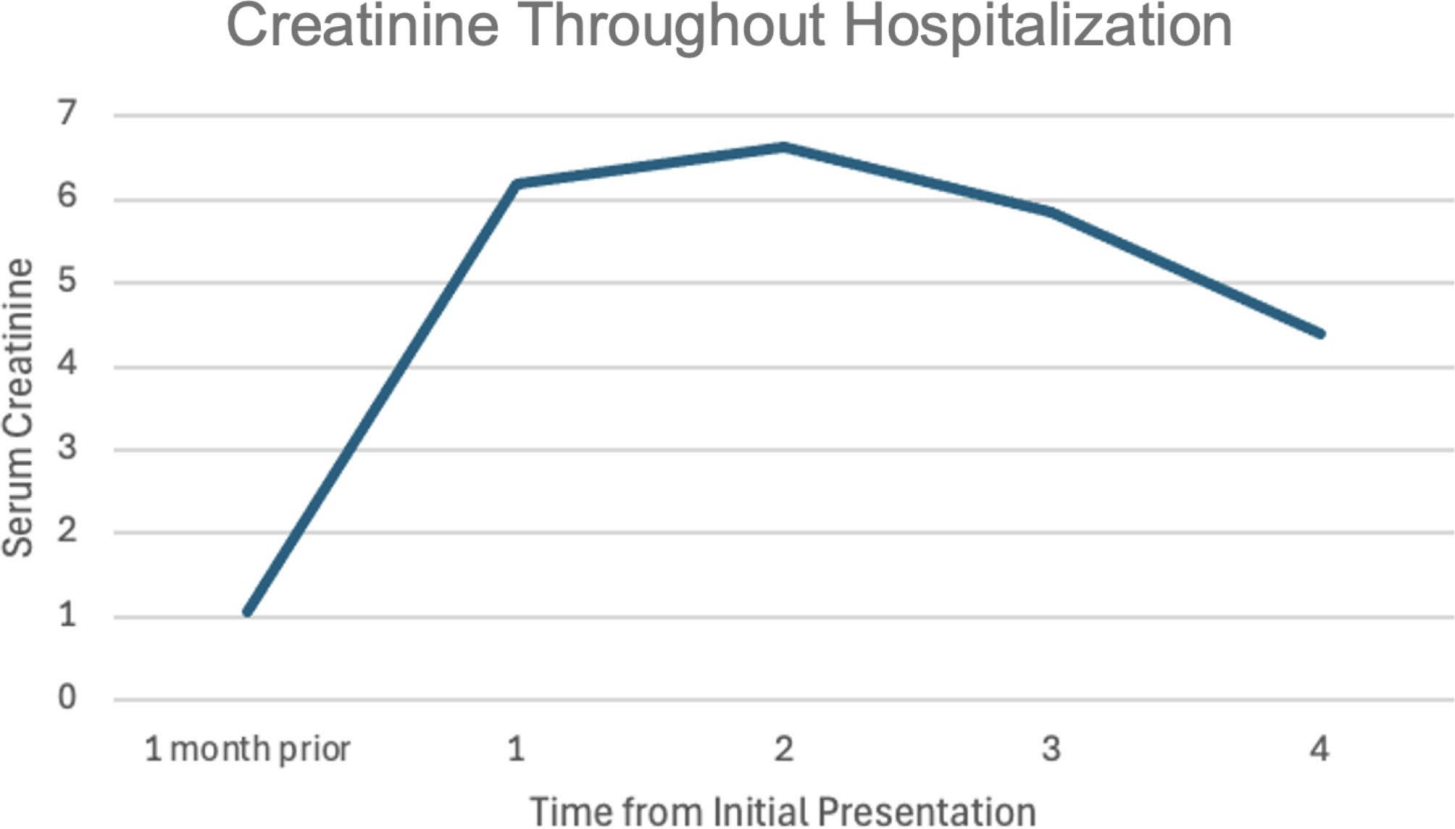
Creatinine trend from a week prior to hospitalization and throughout hospitalization

During her hospital course, her creatinine up trended to 6.63 mg/dL, compared to her baseline of 1.07 mg/dL one month prior (Fig. 1). A renal ultrasound showed a normal right kidney and bladder. No serum or urine eosinophils were found. Fractional excretion of sodium was 2.1%, which pointed to an intrinsic cause of acute kidney injury likely secondary to crystal nephropathy in the setting of valacyclovir use. Intravenous fluids were not given to the patient due the intrinsic etiology and the patient appeared euvolemic on physical exam. Nephrology was consulted due to worsening kidney function on the second day of hospitalization. Nephrology recommended 1300 g of sodium bicarbonate three times a day, and to continue to hold valacyclovir while monitoring the creatinine. Renal biopsy was not done as the patient had a single kidney. In terms of her mental status, the patient stopped responding to internal stimuli by the second day of hospitalization and no longer had any visual hallucinations. On the third day of hospitalization, the patient’s creatinine began improving, and continued down trending to 4.38 mg/dL with strict input and output to ensure the patient had appropriate urinary output. The patient had approximately 1.5–2 L of urinary output daily while in the hospital. Nephrotoxic agents were held throughout hospitalization. Due to the improving creatinine level, the patient was medically stable for discharge and was instructed to follow up with her primary care physician within one week of discharge.

## Discussion

Valacyclovir is an antiviral drug used often for the management of herpes simplex and herpes zoster viruses due to its higher bioavailability. Reported side effects of valacyclovir, as described in various case reports by Abuhelwa et al. 2022, Kato et al. 2022, Murakami et al. 2018, and Zhang et al. 2016 [1, 2, 5, 6] with the most common being acute kidney injury and more rare reports including neurotoxicity. However, the side effects of valacyclovir are not very well recognized by clinicians as there are less reported side effects compared to acyclovir [6].

There have been only two large studies that characterize valacyclovir toxicity. A paper done in Japan by the Pharmaceutical and Medical Device Agency (PMDA) discusses acute kidney injury in patients taking valacyclovir [7]. The paper characterizes patients who developed AKI after taking valacyclovir. Of the 250,000 reported adverse drug reactions using valacyclovir, 514 individuals had concerns of kidney related adverse events. Out of the 514 kidney related adverse events, 344 had AKI. AKI was more common in females older than 40 years. 74.1% did not use any other drugs when taking valacyclovir. Those who took other drugs included valsartan and diclofenac, which contributed to a small portion of AKI. Another study by Lam et al. 2013 in Ontario, Canada discusses risk factors for development of AKI in patients on acyclovir or valacyclovir [3]. The relative risk (RR) of AKI include 4.02 in patients with chronic kidney disease, 1.39 in patients with congestive heart failure, 1.51 in patients with diabetes mellitus, 1.68 in patients taking potassium sparing diuretics, 1.85 in patients using loop diuretics, and 1.39 in patients taking angiotensin receptor blockers (ARBs) or angiotensin converting enzyme (ACE) inhibitors.

There have been many proposed mechanisms for the cause of AKI in patients taking valacyclovir. The most common mechanism agreed by various papers include crystal nephropathy causing obstructive and cellular necrosis [1–3, 6]. Another mechanism involves acyclovir aldehyde effect on tubular dysfunction. Valacyclovir causes direct cell damage, leading to membrane permeability. Destruction of mitochondria inhibits key enzymatic activity leading to cell necrosis [6].

The patient described in our case studies had multiple risk factors that increased her risk of developing AKI. These factors were underlying CKD, diabetes, and usage of ACE inhibitors. This combined effect in addition to a history of underlying nephrectomy caused the patient to develop risk factors for acute renal failure. Our case is unique in that it is the first paper to date that describes a patient with a history of nephrectomy and use of valacyclovir that developed acute renal failure with neurotoxicity. Our patient fits the demographic of an elderly female patient prone to valacyclovir-induced nephrotoxicity [7]. To rule out other etiologies of acute kidney injury and allow for definitive diagnosis, a kidney biopsy should be performed. Kato et al. describes renal biopsy findings in an elderly woman with nephrotoxicity and neurotoxicity induced by valacyclovir [2], which includes acute tubular injury with eosinophilic material in the tubular lumen as well as arteriosclerosis, without glomerulonephritis or interstitial nephritis. However, a limitation in our case was the inability to obtain a renal biopsy due to the patient’s previous history of nephrectomy. Since the 1950s, renal biopsy of solitary kidneys has been contraindicated due to risk of injury, leading to possible nephrectomy [8].

Another less common side effect caused by valacyclovir is neurotoxicity, described by multiple case reports. It was first described by Linssen-Schuurmans in 1998 [9]. Brandariz-Nunez et al. 2021 did a large systematic review to better understand patients who had valacyclovir-induced neurotoxicity [10]. Common manifestations of valacyclovir-induced neurotoxicity include confusion, hallucination, tremors, and dizziness [10, 11]. Risk factors that predisposes a patient to neurotoxicity while on valacyclovir include acute renal failure, high dose of valacyclovir, and age. Neurotoxicity usually manifests within 24–72 h of taking valacyclovir and completely resolves within 2 to 7 days of cessation [12] as seen with our patient. The range in days for resolution of symptoms essentially depends on renal function. To reach a diagnosis of valacyclovir induced neurotoxicity, herpes simplex virus (HSV) encephalitis should first be ruled out. HSV encephalitis is characterized by insidious onset, presence of fever, neck pain, and headache [9, 11]. Our patient presented with nonspecific symptoms consistent with valacyclovir toxicity that had an abrupt onset and without signs of fever, headache, or meningeal irritation. The patient’s clinical improvement of both her AKI and encephalopathy two days after discontinuing the drug also favors our diagnosis of valacyclovir-induced neurotoxicity.

The combination of valacyclovir induced nephrotoxicity and neurotoxicity concomitantly has been rarely described [1]. There have been three papers showing simultaneous presentation of renal injury and encephalopathy induced by valacyclovir use: Murakami et al. 2018, Kenzaka et al. 2021, Abuhelwa et al. 2022 [1, 5, 11]. In these case reports, cessation of encephalopathic symptoms occurred within days after valacyclovir was discontinued and as renal function improved. As in our patient, advanced age was a common factor contributing to the AKI and encephalopathy in all three case reports. Furthermore, the use of ARBs is another risk factor that contributed to AKI in Murakami et al., as in our patient, who used an ACE inhibitor, which has a similar mechanism of ARB, and a contributing factor to AKI. The patient discussed in Abuhelwa et al. had diabetes, which is similar to our patient, a risk factor for AKI. In Kenzaka et al., the patient received 300 mg per day of valacyclovir. However, in Murakami et al. and Abuhelwa et al., the patient received the same dosage as our patient discussed in the case report, 1 gram three times a day. Even at low dosage of 300 mg per day, neurotoxicity and nephrotoxicity was achieved. Our patient should be added to the few case reports of both valacyclovir induced nephrotoxicity and neurotoxicity to further help facilitate a more accurate and timely diagnosis, especially since it has never been described in an individual with a unilateral kidney.

To date, there have been no case reports discussing patients who have had nephrectomy and with concurrent use of valacyclovir, which may have exacerbated our patient’s symptoms. Although the absolute contraindication for renal biopsy in patients with a solitary kidney makes acyclovir-induced nephropathy difficult to definitively diagnose, especially in patients who present with acute renal failure and encephalopathy, a thorough medication review is critical. In summary, patients with a unilateral kidney who present with an AKI and/or altered mental status on valacyclovir should raise prompt concern for drug induced adverse effects, as they might be more susceptible to having them. These patients should be monitored for recovery of renal function. Future large retrospective studies or meta analysis should help identify if there is a dose-dependent effect with use of valacyclovir toxicity.

## Data Availability

No datasets were generated or analysed during the current study.

## Abbreviations

TTP: Thrombotic thrombocytopenic purpura
AKI: Acute kidney injury
CKD: Chronic kidney injury
BUN: Blood urea nitrogen
eGFR: Estimated glomerular filtration ratio
CT: Computed tomography
PMDA: Pharmaceutical and Medical Device Agency
RR: Relative risk
ACE: Angiotensin converting enzyme
HSV: Herpes simplex virus
